# The erythrocyte sedimentation rate and other markers of inflammation in cats tested for *Leishmania infantum* and feline immunodeficiency virus antibodies

**DOI:** 10.1186/s13071-024-06396-1

**Published:** 2024-07-30

**Authors:** Giulia Donato, Tiziana Caspanello, Alessandra Caprì, Massimo De Majo, Nicola Maria Iannelli, Flavia Rosace, Federica Bruno, Germano Castelli, Maria Grazia Pennisi, Marisa Masucci

**Affiliations:** 1 Università di Messina, Messina, Italy; 2 ASC “I Periodeuti”, Reggio Calabria, Italy; 3 Clinica Veterinaria Camagna–VetPartners, Reggio Calabria, Italy; 4 National Reference Center for Leishmaniasis (C.Re.Na.L.), Istituto Zooprofilattico Sperimentale della Sicilia, Palermo, Italy

**Keywords:** Erythrocyte sedimentation rate, Acute phase proteins, Cat, Leishmaniosis, FIV, Co-infection

## Abstract

**Background:**

In endemic areas, *Leishmania infantum* and feline immunodeficiency virus (FIV) co-infection occurs in cats, and may favour a progressive course of feline leishmaniosis. Abnormalities in serum protein fractions have been reported, but inflammation markers have scarcely been studied. Erythrocyte sediment rate (ESR) is a marker of inflammation that is poorly used in veterinary medicine, but it has been evaluated in EDTA blood using a recently introduced automatic device. We studied ESR and a pool of feline markers of inflammation (MoI) in cats *L. infantum* (*Li*^+^) and/or FIV antibody-positive (*Li*^+^FIV^+^/FIV^+^) with the aims (a) to evaluate ESR as MoI in cats with the infectious and clinical conditions considered and (b) to provide data about a pool of MoI never investigated in the feline infections studied and in other cat diseases before.

**Methods:**

This prospective controlled study included 35 study group cats (*Li*^+^, *n *= 20; FIV +, *n* = 8; *Li*^+^FIV^+^, *n* = 7) and ten healthy antibody-negative control cats. Clinical findings at physical examination and selected clinical pathological abnormalities related to inflammation were statistically analysed in relation to the infectious status and ESR values.

**Results:**

ESR values were higher in *Li*^+^, FIV^+^, and *Li*^+^FIV^+^ cats compared with control cats, and 40% of the study group cats had ESR values above the reference interval (RI). ESR positively correlated with some positive MoI and negatively with some negative MoI studied. Additionally, a higher prevalence of ESR values above the RI has been detected in cats with hypoalbuminemia or hypergammaglobulinemia and higher ESR values were measured in cats with serum protein electrophoresis (SPE) fraction abnormalities. Correlations were also found with erythrocytes, hemoglobin, hematocrit and some erythrocyte indices. FIV^+^ and *Li*^+^FIV^+^ cats had a higher prevalence of increased ESR values, and almost all had SPE abnormalities and more severe clinical presentations compared with *Li*^+^ cats.

**Conclusions:**

Abnormal levels of MoI were found in almost all parameters studied, particularly in FIV^+^ and *Li*^+^FIV^+^ cats. Also, ESR can be used as a marker of inflammation in cats with *L. infantum* and/or FIV infection.

**Graphical Abstract:**

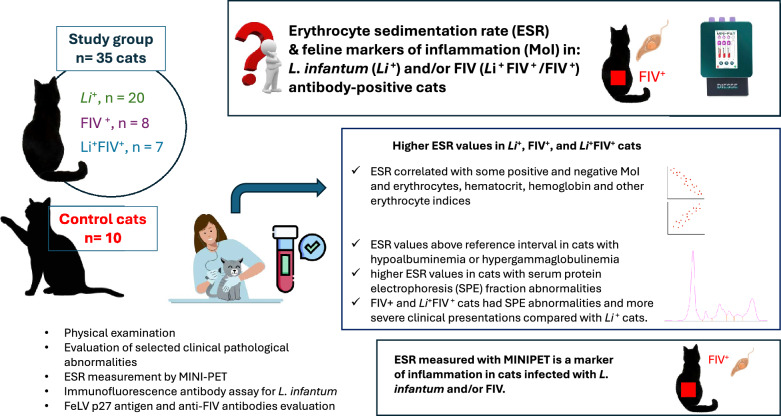

**Supplementary Information:**

The online version contains supplementary material available at 10.1186/s13071-024-06396-1.

## Background

Leishmaniosis caused by *Leishmania infantum* is a zoonotic disease affecting mainly dogs in endemic areas of the Mediterranean Basin and the Americas, and dogs are also considered the main domestic reservoirs of the parasite [1]. Leishmaniosis is less frequently observed in cats; however, it is considered an emerging feline disease [2]. The course of *L. infantum* infection in dogs is influenced by the host immune response, with progressive infection and development of disease when the parasite induces a marked humoral immune response and downregulates cell-mediated immunity [3, 4]. *Leishmania infantum*-specific production of antibodies and IFN-γ have been reported in cats exposed to the parasite in endemic areas [5]. Similarities in the immunopathogenesis of *L. infantum* infection have been observed in a study comparing cats and dogs, but cats showed a lower level of both humoral and cell-mediated immune response [6]. Feline immunodeficiency virus (FIV) co-infection may play a role as a risk factor for feline leishmaniosis (FeL), and significant associations between the two pathogens have been reported [7–12].

Although dermatological lesions are a frequent reason for veterinary consultation of cats with FeL [13, 14], systemic signs and clinical pathological abnormalities are usually detected because the disease is associated with a generalised infection [13]. Mild-to-moderate, non-regenerative anaemia and proteinuria are common findings, while other cell blood count (e.g. pancytopenia, thrombocytopenia) and serum biochemical (e.g. hypoalbuminaemia, increased liver enzymes, azotaemia) changes are rarer [13]. Complete blood count (CBC) changes are known to occur in inflammatory diseases and in endemic areas, some of these CBC abnormalities were more frequently observed in *L. infantum*-positive cats and in cats co-infected with FIV [15]. Hyperproteinaemia with hyperglobulinaemia and hypergammaglobulinaemia are the most prevalent serum abnormalities [13]. Some studies investigated the electrophoretic fractions of serum proteins in cats and reported increases of the α_2_, β_2_ and γ-globulins with polyclonal gammopathies in *L. infantum*-infected cats [16, 17]. Changes in total proteins and electrophoretic protein fractions are routinely evaluated and monitored in *L. infantum*-infected dogs, where hypoalbuminaemia, increases of α_2_-globulins and γ-globulins can be observed when progression to disease occurs [18–22]. An intense inflammatory response is generally observed in leishmaniotic dogs, and abnormalities in various acute-phase proteins have been reported in their clinical pathological evaluation [19]. Serum amyloid A (SAA) is a feline major positive acute phase protein (APP) as the concentration increases rapidly and significantly (10–100 times) in serum in case of proper stimulation, and it also decreases fast [23]. SAA is the main feline marker of acute inflammation, and it is usually included in the routine biochemical profile of cats; however, it has been scarcely investigated in cats with FeL. In a recent case of FeL with ulcerative dermatitis and chronic uveitis, increased values of SAA have been reported at diagnosis and changes of concentration have been considered useful for monitoring the course of disease [24]. Two studies included SAA in the clinical pathological investigations of cats tested for *L. infantum* and positive cats did not show higher values compared with healthy control cats. There are no publications evaluating other APPs in cats with *L. infantum* infection or disease. However, α-1-acid glycoprotein (AGP) and haptoglobin (Hp) are positive moderate APPs (i.e. increasing less rapidly, < tenfold and decreasing slower than major APPs) investigated in various infectious and non-infectious feline diseases [23]. In particular, AGP is considered a specific clinical pathological marker of feline infectious peritonitis [23]. Albumin and total iron binding capacity (TIBC) concentrations are defined as negative APPs as they decrease in case of inflammation, and they do it slowly [23]. Similarly, serum iron is a negative marker of inflammation [25], and it is also reduced in case of iron depletion (e.g. in chronic haemorrhage), but in this latter case, a normal-to-increased value of TIBC is found while in inflammatory conditions TIBC value is normal-to-decreased [26]. As both causes of low iron levels induce non-regenerative anaemia, the simultaneous evaluation of iron and TIBC contributes to differentiating anaemia of inflammatory disease from anaemia of iron deficiency [26].

In recent years, blood cell ratios (BCRs) (NL, neutrophil-to-lymphocyte; ML, monocyte-to-lymphocyte; PL , platelet-to-lymphocyte ratios) have been investigated as cost-effective and easily accessible diagnostic and prognostic markers of phlogistic and neoplastic [27, 28] diseases has been proposed in cats. The relationships between NL, ML and PL ratios and some markers of inflammation routinely measured in cats (SAA, albumin, globulin, albumin-to-globulin ratio and leukocyte alterations suggestive of inflammation) have been investigated, and authors concluded that these BCRs are good markers of inflammation in cats [29]. However, no studies have currently evaluated their changes in FeL, FIV and feline leukaemia virus (FeLV) infections or co-infection in cats.

The erythrocyte sedimentation rate (ESR) is an old marker of inflammation, and the test measures in citrated blood the distance (mm/h) that red blood cells (RBC) cover while they sediment inside a vertical graduated test tube. The Westergren assay is the gold standard method for performing ESR measurements in humans and animals. Westergren method has been neglected in veterinary medicine, particularly in cats because of the limitation of dedicating 1 mL of blood to this test [30–32]. New automatic technologies have been developed in human medicine with the aim to perform ESR in laboratories managing high volume of samples, to speed up the test and to avoid any withdraw of blood from sample tubes for maximum user safety. In some cases, the new technologies validated the use of K_3_ EDTA blood instead of citrated blood, and this latter characteristic opened the possibility of measuring ESR with the same EDTA blood tube used for CBC. In 2020, a new automated portable ESR device operating with the K_3_ EDTA tubes used for CBC has been marketed for measuring ESR in dogs, cats and horses (MINI-PET, DIESSE Diagnostica Senese, Monteriggioni, Siena, Italia). The instrument has been evaluated in dogs [32–34], horses [35] and cats [36, 37]. The two studies performed in cats investigated the effects of some analytical (test duration) and blood sample (packed cell volume, temperature and length of storage of blood) variables on the ESR values [36] and the changes observed in feline chronic kidney disease [37]. Cavalera et al. [34] measured the ESR in dogs with canine leishmaniosis (CanL) with MINIPET and reported increased values in dogs affected by active form of disease compared with exposed or healthy dogs [34].

This prospective study assessed values of ESR and of other markers of inflammation in clinically evaluated cats with antibody titres positive to *L. infantum* (*Li*^+^), FIV (FIV^+^) and both pathogens (*Li*^+^ FIV^+^), compared with healthy antibody-negative cats, and investigated correlations between their values. The aims were: (a) to test the hypothesis that ESR values measured in EDTA blood with an automated point-of-care (POC) device that can be used as a marker of inflammation in cats with the infectious and clinical conditions considered and (b) to provide data about a pool of markers of inflammation never investigated in the feline infections studied and in other cat diseases before.

## Methods

### Study sites, cat enrollment and sampling procedures, and study groups

From March to December 2023, cats that presented for annual health checks, minor medical problems, elective, minor or orthopedic surgeries, referred to the Clinica Veterinaria Camagna-VetPartners (Reggio Calabria, Italy) were prospectively evaluated. The study protocol has been approved by the ethics committee of the Department of Veterinary Sciences of the University of Messina (no. 05/23), and the cat owners signed the informed consent form, which provided relevant information about the study protocol.

Cats older than 6 months and irrespective of sex, weight, breed, reproductive and clinical status were evaluated. Exclusion criteria included: age below 6 months, critical clinical conditions, diagnosis of neoplasia, endocrine, allergic and lungworm disease, feline infectious peritonitis, severe acute upper respiratory tract disease, feline panleukopaenia, serum positivity to FeLV-p27 antigen rapid test, and treatments with at least one of the following drugs in the last 30 days: corticosteroids, immunosuppressive and non-steroidal anti-inflammatory drugs. Demographic data (i.e. breed, age and sex), baseline information (i.e. clinical history, concomitant treatments and previous diseases), physical examination findings and any diagnosis of comorbidities were recorded for each cat. A total of 3–5 mL of blood were taken from each cat: 1 mL was placed into a K_3_ EDTA tube and used for ESR and CBC evaluations. The remaining blood was used to perform blood smears and to obtain serum after clotting in a plain tube and centrifugation for biochemical, electrophoretic and antibody testing. In the case of cats admitted for elective, minor or orthopaedic surgeries, blood collection was performed before the surgical procedures. Urine samples (about 5 mL) were obtained by cystocentesis or free catch and used for urinalysis and urine protein to creatinine ratio (UPC) evaluations.

The study group included cats with various clinical and/or clinical pathological abnormalities and resulting antibody positive to *L. infantum* and/or FIV, and negative for FeLV. Indoor, client-owned cats that were clinically healthy according to history, physical examination and clinical pathological evaluation, and antibody negative to *L. infantum*, FIV and FeLV infections were enrolled as a control group.

### Clinical pathological and ancillary investigations

The CBC was performed using the haematology analysers Siemens Advia 2120 (MyLav—La Vallonea, Laboratorio di Analisi Veterinarie s.r.l., Milan, Italy), ProCyte Dx (IDEXX laboratories, Westbrook, ME, USA) or Futurlab DF50 (Padova, Italy). The absolute counts of neutrophils, monocytes and platelets were divided by the absolute count of lymphocytes and NL, ML and PL ratios were calculated [29]. The platelet counts were excluded, and the PL ratio was not calculated when platelet clumps were detected in blood smears.

The ESR measurement was performed using an automated POC device following the procedure provided by the manufacturer (MINI-PET, DIESSE, Diagnostica Senese S.p.A., Siena, Italy). A serum biochemical profile (SBP) was performed with the Beckman Coulter AU 5800 analyser (MyLav—La Vallonea, Laboratorio di Analisi Veterinarie s.r.l., Milan, Italy). Serum protein electrophoresis (SPE) was performed with the capillary electrophoresis system Sebia Capillarys 3 Tera (MyLav—La Vallonea, Laboratorio di Analisi Veterinarie s.r.l., Milan, Italy) [38].

Urinalysis was made with the analyser Roche Cobas U601 and the UPC was analysed with Beckman AU640 (MyLav—La Vallonea, Laboratorio di Analisi Veterinarie s.r.l., Milan, Italy).

The evaluated parameters' reference intervals (RI) were internally estimated by MyLav—La Vallonea, Laboratorio di Analisi Veterinarie s.r.l., Milan, Italy, and are reported in a Supplementary Table (Table S1) [39]. In case of ESR, the RI considered was within values of 2.5th and 97.5th percentile of control group cats (1–48 mm/h).

Due to technical reasons, insufficient volume of serum and the lack of urine samples, not all parameters of SBP, SPE and urinalysis tests were evaluated in all cats, as shown in Table S1.

Routine diagnostic investigations were additionally performed in study group cats to rule out comorbidities based on a problem-oriented medical approach. In particular, fine-needle insertion (FNI) was performed in case of enlarged lymph nodes and stained lymph node smears (May Grünwald-Giemsa stain, Merck KgaA, Darmstadt, Germany) were cytologically evaluated [40].

### ***L. infantum***, FIV, and FeLV investigations

Anti-*L. infantum* antibodies were detected by immunofluorescence antibody assay (IFA). Antigen slides were produced by C.Re.Na.L. (Centro di Referenza Nazionale per la Leishmaniosi, Palermo, Italy) using *L. infantum* strain MHOM/IT/80/IPT1. A fluorescent anti-cat immunoglobulin G (IgG) antibody [anti-feline IgG (H + L)-FITC, Fuller Laboratories, Fullerton, CA, USA] was used according to Persichetti et al. [29, 40]. The cut-off value for positivity was set at 1:80 [41, 42]. FeLV p27 antigen and anti-FIV antibodies were evaluated in serum samples by the SensPERT™ FeLV Ag/FIV Ab test kits (VetAll, Goyang, Korea) and results were confirmed by ELISA (FIVCHECK Ab ELISA and FeLVCHECK Ag ELISA, Agrolabo spa, Milano, Italia) (MyLav—La Vallonea, Laboratorio di Analisi Veterinarie s.r.l., Milan, Italy).

### Statistical analysis

Statistical analysis was performed using Jamovi 2.3.28 statistical software program.

The distribution of continuous variables was evaluated by the Shapiro–Wilk test. Spearman’s Rho test was used to evaluate the correlation between ESR and the other markers of inflammation. The strength of this relationship, according to the correlation coefficient absolute value (*r*_s_), was qualified as follows: *r*_s_ = 1: perfect correlation; 1 > *r*_s_ ≥ 0.8: strong correlation; 0.8 > *r*_s_ ≥ 0.4: moderate correlation; 0.4 > *r*_s_ > 0: weak correlation; and *r*_s_ = 0: no correlation [43]. The critical value of *r*_s_ was established on the basis of the number of pairs of scores for each pair of parameters evaluated [44]. Mann–Whitney *U* test was used to compare age between the control and study groups and to compare the values of ESR and other markers of inflammation between (a) control and study groups, (b) control group and *Li*^+^ cats, (c) control group and FIV^+^ cats, and (d) control group and *Li*^+^ FIV^+^ cats. Fisher’s exact test was used to compare differences in sex between the study group and control group and to evaluate the association between serological status and clinical signs and parameters with outlier values. Differences were considered significant if *P* values were < 0.05.

## Results

### Cats studied and their serological, clinical and clinical pathological evaluations

A cohort of 45 cats was enrolled, and 35 cats were included in the study group, while 10 cats formed the control group. In the study group of cats (21 males and 14 females; mean age 54.5 ± 49.6 months), 17 cats were indoors, 15 were outdoors, and three cats lived both indoors and outdoors. Control group cats (seven females and three males; mean age 26 ± 35.3 months) were all indoors. No significant differences were found between the two groups concerning age and sex.

Among the 35 cats in the study group, 20 were *Li*^+^, 8 were FIV^+^, and 7 were *Li*^+^ FIV^+^. The *L. infantum* IFA titres (*n* = 27 cats) ranged from 1:80 to 1:320 dilutions (1:80, *n* = 19; 1:160, *n* = 6; 1:320, *n* = 2).

Abnormalities were observed at physical examination (65.7%; 23/35) and the clinical pathological evaluation (94.3%; 33/35) of the study group cats, and they are described as individual cats with their antibody-positive status in Table 1. Various oral disorders (periodontitis, *n* = 9; stomatitis, *n* = 5; dental calculi, *n* = 4; tongue ulcers, *n* = 1) were found. Enlarged lymph nodes (*n* = 11) was the more prevalent clinical sign and lymphoid hyperplasia was cytologically diagnosed. Crusty dermatitis (*n* = 2, with alopecia and pruritus in one case), alopecia (*n* = 2) and squamous dermatitis with alopecia (*n* = 1) were the dermatological lesions detected. Conjunctivitis (*n* = 3) and keratoconjunctivitis (*n* = 1) were the ophthalmic findings observed. Rhinitis (*n* = 1) and diarrhoea (*n* = 1) affected one single cat each. Flea infestation was detected in one cat, but it was not associated with clinical manifestations.

**Table 1:** Clinical and clinical pathological abnormalities and ESR values in the study group cats based on their antibody positivity to *L. infantum* and/or FIV

Cat number	*L. infantum* IFA titre	Clinical findings	CBC	SBP	SPE	ESR	Urinalysis with UPC
*L. infantum*							
1L	80	NTR	Eosinopaenia	NTR	↑ γ-globulins	↑	NP
2L	80	Dental calculi, enlarged lymph nodes	NTR	NTR	NTR	(=)	NTR
3L	160	NTR	Mild nr-anaemia	↑ AGP ↑ ALP ↑ P	↑ γ-globulins	↑	NP
4L	80	NTR	Thrombocytopaenia	NTR	↑ α_1_-globulins	(=)	NP
5L	80	Dental calculi	Leukocytosis, neutrophilia, monocytosis	NTR	↑ α_1_ and γ-globulins ↓ A/G	(=)	↓ USG
6L	80	Enlarged lymph nodes, conjunctivitis	NTR	↑ ALT ↑ ALP ↑ P	NTR	(=)	NP
7L	320	NTR	Lymphocytosis	↑ Bilirubin	↑ γ-globulins	↑	NP
8L	160	NTR	Lymphocytosis	NTR	NTR	(=)	NP
9L	160	NTR	NTR	↑ ALT	↑ γ-globulins	↑	Proteinuria
10L	80	NTR	NTR	↓ Fe; ↑ ALP	NTR	(=)	NTR
11L	80	Periodontitis, enlarged lymph nodes	NTR	↑ ALP ↑ P	NTR	(=)	Proteinuria
12L	80	Periodontitis	NTR	↓ Fe	NTR	(=)	NP
13L	80	Periodontitis	NTR	^	NP	(=)	NP
14L	80	NTR	NTR	↑ ALT ↑ ALP	NTR	(=)	NTR
15L	80	Flea infestation	Thrombocytosis	↓ Fe ↑ ALP ↑ TP ↑ P	NTR	(=)	NP
16L	160	NTR	NTR	NTR	↑ γ-globulins	↑	NP
17L	80	Periodontitis	NTR	↑ ALP ↑ P	NTR	(=)	NP
18L	80	Periodontitis	NTR	↑ Hp ↑ ALP ↑ P	NTR	(=)	NP
19L	80	NTR	NTR	↑ SAA ↑ ALT ↑ P	↑ α_1_-globulins	(=)	NP
20L	80	Stomatitis	NTR	↓ Fe ↑ ALP	NTR	↑	NP
FIV							
1F	NA	Enlarged lymph nodes, conjunctivitis, pruritic crusty alopecic dermatitis	NTR	NTR	↑ γ-globulins	↑	NTR
2F	NA	Enlarged lymph nodes, conjunctivitis, alopecia, stomatitis, diarrhoea	NTR	↑ SAA ↑Hp	↑ γ-globulins	(=)	NP
3F	NA	NTR	NTR	↑ glucose∞	NTR	(=)	NTR
4F	NA	Dental calculi	Moderate nr-anaemia	↓ Fe ↑ BUN ↑ Cr	↑ α_1_-globulins ↑ γ-globulins	↑	↓ USG, proteinuria
5F	NA	Enlarged lymph nodes, dental calculi, periodontitis, squamous alopecic dermatitis	NTR	↑ SAA ↑ Hp ↑ ALT ↑ Bilirubin ↑ glucose∞	↓ albumin ↓ A/G ↑α_1_-globulins ↑ α_2_-globulins↑ γ-globulins	↑	Proteinuria
6F	NA	Crusty dermatitis	NTR	NTR	↑ α_1_-globulins ↑ γ-globulins	(=)	NTR
7F	NA	Enlarged lymph nodes, periodontitis	NTR	↓ Fe	↓ albumin ↓ A/G ↑ α_1_-globulins ↑ γ-globulins	↑	NTR
8F	NA	Keratoconjunctivitis	NTR	NTR	↑ α_1_-globulins	↑	NTR
FIV and *L. infantum*							
1LF	80	Enlarged lymph nodes, rhinitis	NTR	↑ IgM	↑ γ-globulins	↑	NTR
2LF	320	Stomatitis, tongue ulcers	NTR	↓ Fe, ↑ SAA ↑ Hp	↓ albumin ↑ globulins ↑ γ-globulins ↓ A/G	↑	NTR
3LF	80	Enlarged lymph nodes, periodontitis, alopecia	NTR	NTR	↑ γ-globulins	(=)	NTR
4LF	80	Enlarged lymph nodes, periodontitis	Mild nr-anaemia, eosinophilia	↓ Fe, ↑ SAA	↑ γ-globulins	(=)	Proteinuria
5LF	80	Enlarged lymph nodes, stomatitis	NTR	↑ IgM ↑ Hp ↑ BUN ↑ Cr ↑ SDMA ↓ Fe	↓ albumin ↓ A/G ↑ globulins ↑ α_2_-globulins ↑ γ-globulins	↑	↓ USG
6LF	160	NTR	Lymphopoenia	↑ IgM ↑ ALT ↑ glucose ∞	↑α_1_-globulins	(=)	NTR
7LF	80	Stomatitis	NTR	↑ SAA	↑ γ-globulins	(=)	NTR

CBC, complete blood count; SBP,  serum biochemical profile; SPE, serum protein electrophoresis; UPC , urinary protein-to-creatinine ratio; NTR, nothing to report; NP, not performed; nr-anaemia, non-regenerative anaemia; AGP, α-1 acid glycoprotein; ALP, alkaline phosphatase; ALT, alanine aminotransferase; P, phosphorus; A/G, albumin-to-globulin ratio; USG, urine specific gravity; Fe, iron; ^ evaluated only SAA, Hp and AGP; TP, total proteins; Hp, haptoglobin; SAA, serum amyloid A; BUN, blood urea nitrogen; Cr, serum creatinine; SDMA, serum symmetric dimethylarginine; ↑ value above reference interval; ↓ value below reference interval; ( =)  ESR value ≤ 48 mm/h; ∞ fructosamines within the reference interval (stress hyperglycaemia)

Prevalence of clinical signs and lymph node enlargement was lower in *Li*^+^ cats compared with FIV^+^ and *Li*^+^ FIV^+^ cats [*P* = 0.034, odds ratio (OR) = 6.5, 95% confidence interval (CI) = 1.082–33.11; *P* = 0.0271, OR = 6.476, 95% CI = 1.39–26.23, respectively]. The most prevalent clinical pathological abnormality was the increase of γ-globulins (*n* = 18) and polyclonal gammopathies were observed. Increases of α_1_ (*n* = 9) and α-_2_ (*n* = 2) globulins were seen and associated with polyclonal gammopathy, respectively, in five and one cats. A low value of Fe was found in nine cats, and two out of the three cats with anaemia (normocytic normochromic, non-regenerative; two mild and one moderate) had low Fe concentrations (4F, 4LF), as well as the unique cat with thrombocytosis (15L). Low A/G values were detected in five cats, four of which had hypoalbuminaemia, and this was associated with increased concentrations of Hp, SAA or IgM or low Fe measures. In the SBP and urinalysis, the more prevalent abnormalities were values above the RI of ALP (phosphatase alkaline) (*n* = 9), P (*n* = 7), ALT (alanine  aminotransferase) (*n* = 6), SAA (*n* = 6), Hp (*n* = 5) and proteinuria (*n* = 4). Other parameters were increased in a lower number of cats: IgM (*n* = 3), glucose (*n* = 3), BUN (blood urea nitrogen) (*n* = 2), Cr (creatinine) (*n* = 2), bilirubin (*n* = 2), SDMA (symmetric dimethylarginine) (*n* = 1) and AGP (*n* = 1). Urinary specific gravity was inappropriate in three cats. White blood cell abnormalities were rare: lymphocytosis (*n* = 2), leukocytosis with neutrophilia and monocytosis (*n* = 1), eosinophilia (*n* = 1), eosinopaenia (*n* = 1) and lymphopaenia (*n* = 1). Thrombocytopaenia was observed in one cat only.

### Values of ESR and data analysis of the markers of inflammation

Descriptive statistics of values of ESR and the other markers of inflammation considered are reported in Table 2.

**Table 2:** Median, minimum (min), maximum (max), 25–75th percentile of control group (*n* = 10) and study group (*n* = 35), consisting of *L*. *infantum* (*Li*^+^, *n* = 20) and FIV (FIV^+^, *n* = 8) positive cats or co-infected cats (*n* = 7) for the ESR and other markers of inflammation statistically evaluated

Parameter (reference unit)	Control group Median (min, max) [25th–75th]	Study group Median (min, max) [25th–75th]	*Li*^+^ Median (min, max) [25th–75th]	FIV^+^ Median (min, max) [25th–75th]	*Li*^+^ and FIV^+^ Median (min, max) [25th–75th]
ESR (mm/h)	13.5 (1.0–48.0) [9.8–24.0]	*37.0 (10.0–71.0) [19.0–56.5]	*29.5 (10.0–61.0) [15.3–50.0]	*55.5 (13.0–66.0) [22.5–58.8]	*45.0 (15.0–71.0) [34.5–66.5]
Complete blood count					
Red blood cells (M/μL)	9.07 (6.24–11.50) [7.32–9.91]	7.58 (4.13–10.50) [6.68–9.09]	7.88 (5.61–10.50) [6.89–9.26]	6.89 (4.13–9.11) [6.36–7.84]	7.90 (6.16–9.81) [6.49–8.88]
Mean corpuscular volume (fL)	41.0 (38.8–46.4) [39.7–43.3]	41.2 (31.8–53.6) [38.8–44.5]	39.7 (31.8–53.6) [38.1–42.9]	44.8 (36.4–47.7) [42.5–45.0]	40.2 (32.0–44.2) [39.2–41.6]
Haemoglobin (g/dL)	12.4 (8.7–15.3) [9.9–13.8]	10.5 (5.9–13.7) [9.4–11.7]	10.8 (7.1–13.7) [10.0–11.8]	9.9 (5.9–13.2) [9.6–10.8]	8.9 (8.2–13.1) [8.7–11.5]
Haematocrit (%)	36.4 (27.0–44.5) [31.2–41.4]	*31.3 (18.5–42.7) [28.8–34.4]	32.4 (22.4–42.7) [29.4–34.8]	30.4 (18.5–40.9) [28.1–31.6]	29.0 (26.2–38.7) [27.0–33.4]
MCHC (g/dL)	32.5 (31.6–35.6) [32.3–34.3]	33.5 (28.4–37.3) [32.1–34.7]	33.3 (28.5–37.3) [32.5–34.6]	33.5 (31.0–35.7) [32.1–33.5]	33.5 (28.4–34.6) [32.4–34.0]
White blood cells (K/μL)	8.92 (4.52–13.80) [5.53–10.60]	*11.50 (5.03–24.20) [8.82–14.80]	*11.70 (7.75–24.20) [9.21–15.60]	11.80 (6.66–17.20) [8.40–13.00]	10.00 (5.03–18.60) [6.91–14.50]
Neutrophils (K/μL)	4.27 (1.58–10.94) [2.90–5.73]	*6.53 (2.86–15.95) [5.17–9.17]	*6.65 (2.96–15.95) [5.48–8.99]	5.61 (3.95–12.44) [4.88–8.08]	7.56 (2.86–12.16) [4.33–9.63]
Lymphocytes (K/μL)	2.71 (1.27–4.40) [2.37–3.55]	3.12 (0.27–7.97) [2.08–4.36]	3.37 (1.10–7.97) [2.42–5.95]	3.26 (1.41–6.16) [2.55–4.16]	1.90 (0.27–3.91) [1.54–3.16]
Monocytes (K/μL)	0.18 (0.04–0.70) [0.11–0.24]	0.25 (0.00–1.04) [0.18–0.42]	0.25 (0.00–1.04) [0.17–0.41]	0.33 (0.13–0.67) [0.21–0.43]	0.25 (0.08–0.43) [0.18–0.36]
Eosinophils (K/μL)	0.58 (0.29–1.31) [0.41–0.88]	0.77 (0.12–4.14) [0.48–1.21]	0.79 (0.13–1.85) [0.42–1.22]	0.68 (0.31–1.49) [0.46–0.93]	0.70 (0.12–4.14) [0.65–1.12]
Platelets (K/μL)	233 (171–528) [188–252]	284 (133–640) [180–363]	198 (133–640) [167–301]	297 (182–422) [279–373]	341 (142–470) [264–396]
NL	1.6 (0.6–6.5) [1.0–2.1]	2.1 (0.4–14.5) [1.4–3.4]	2.0 (0.4–6.7) [1.2–3.1]	1.4 (0.9–3.4) [1.2–2.3]	*2.9 (1.5–14.5) [2.2–5.8]
ML	0.1 (0.0–0.2) [0.0–0.1]	0.1 (0.0–0.6) [0.0–0.1]	0.1 (0.0–0.4) [0.0–0.1]	0.1 (0.0–0.1) [0.0–0.1]	0.1 (0.0–0.6) [0.1–0.2]
PL	95.1 (56.5–313.0) [80.8–148.0]	96.8 (17.7–582.0) [66.1–131.0]	79.3 (17.7–582.0) [53.6–116.0]	68.5 (59.8–101.0) [64.2–79.1]	135.0 (92.6–522.0) [115–236.0]
Serum biochemical profile					
IgG (mg/dL)	480 (329–860) [468–555]	*802 (287–1110) [612–990]	756 (287–996) [507–849]	*931 (612–1048) [829–1008]	*1043 (573–1110) [925–1058]
IgM (mg/dL)	76 (24–129) [56–80]	94 (39–257) [68–111]	82 (39–142) [67–105]	79 (56–149) [64.5–118]	*111 (53–257) [103–187]
Fe (μg/dL)	83.5 (27.0–123.0) [73.8–91.8]	65.5 (15﻿.0–142﻿.0) [48.8–81.8]	66﻿.0 (44﻿.0–142﻿.0) [55﻿.0–78﻿.0]	79﻿.0 (33﻿.0–106﻿.0) [52﻿.0–89.5]	*55﻿.0 (15﻿.0–86﻿.0) [42﻿.0–67﻿.0]
TIBC (μg/dL)	309 (265–397) [276–333]	289 (201–388) [270–314]	289 (208–369) [271–314]	315 (201–388) [288–350]	271 (251–300) [268–292]
Serum amyloid A (μg/mL)	1.2 (0.1–2.7) [0.1–1.8]	0.1 (0.1–110.0) [0.1–1.8]	0.1 (0.1–9.8) [0.1–0.1]	1.4 (0.1–110.0) [0.1–6.8]	1.1 (0.1–12.6) [0.5–7.8]
Haptoglobin (mg/dL)	52.9 (36.8–93.6) [44.5–65.5]	*98.2 (32.9–541.0) [78.5–123.0]	*95.7 (32.9–514.0) [81.7–118.0]	*93.8 (40.4–200.0) [76.3–123.0]	*106.0 (67.0–204.0) [86.7–159.0]
AGP (μg/mL)	192 (105–297) [136–255]	257 (145–1335) [215–353]	255 (145–912) [203–315]	247 (153–1335) [223–382]	335 (149–513) [232–372]
Total proteins (g/L)	70.45 (61.0–77.7) [64.3–72.9]	*75.7 (62.4–92.0) [72.7–83.0]	72.9 (62.4–85.2) [67.5–75.6]	*79.5 (74.0–86.8) [75.9–82.7]	*83.8 (75.8–92.0) [82.4–89.6]
Serum protein electrophoresis					
Albumin (g/L)	37.3 (28.9–44.4) [32.8–38.6]	33.0 (23.8–43.1) [30.9–35.7]	34.1 (26.9–38.2) [32.6–36.1]	31.3 (25.2–43.1) [28.8–33.9]	30.8 (23.8–40.1) [27.0–33.5]
α_1_-globulins (g/L)	2.7 (1.9–3.1) [2.5–2.9]	*3.2 (1.9–4.7) [2.6–3.6]	3.0 (2﻿.0–4﻿.0) [2.5–3.4]	*3.8 (1.9–4.7) [3.0–4.0]	*3.2 (2.2–3.7) [2.9–3.3]
α_2_-globulin (g/L)	9.9 (8.1–12.6) [8.9–11.9]	10.9 (7.4–15.9) [9.8–11.9]	10.6 (7.4–13.0) [9.3–11.3]	*11.5 (9.7–15.9) [10.7–12.8]	11.0 (9.2–15.1) [10.3–11.8]
β_1_-globulins (g/L)	2.9 (1.8–4.9) [2.7–3.4]	3.6 (1.2–5.5) [2.8–4.1]	3.6 (1.2–5.5) [2.7–3.8]	3.4 (2.3–4.8) [2.7–4.2]	*4.0 (3.2–4.7) [3.7–4.3]
β_2_-globulins (g/L)	2.4 (1.1–4.4) [1.7–3.5]	3.2 (1.0–4.4) [1.8–3.7]	3.2 (1.0–4.4) [1.6–3.3]	3.7 (1.8–4.4) [3.1–4.1]	1.8 (1.6–4.2) [1.7–3.4]
γ-globulins (g/L)	11.8 (7.4–19.9) [9.7–13.4]	*21.6 (8–40.4) [16.2–27.3]	16.9 (8–31.1) [11.7–21.6]	*23.4 (14.4–28.6) [21.5–26.4]	*28.7 (20.3–40.4) [27.2–35.0]
Globulins (g/L)	32.8 (25.6–44.8) [29.9–34.0]	*43.0 (27.6–68.2) [37.9–51.7]	38.5 (27.6–54.4) [33.3–42.1]	*48.9 (34.9–53.7) [43.8–52.2]	*52.5 (42.8–68.2) [49.3–58.1]
A/G	1.13 (0.74–1.58) [0.94–1.24]	*0.80 (0.35–1.34) [0.61–0.97]	0.90 (0.53–1.34) [0.80–1.08]	*0.65 (0.48–1.12) [0.54–0.77]	*0.58 (0.35–0.94) [0.49–0.65]

ESR, erythrocyte sedimentation rate; MCHC, mean corpuscular hemoglobin concentration; A/G, albumin-to-globulin ratio; NL, neutrophil-to-lymphocyte ratio; ML, monocyte-to-lymphocyte ratio; PL, platelet-to-lymphocyte ratio; IgG, immunoglobulins G; IgM, immunoglobulins M; Fe, iron; TIBC, total iron binding capacity; AGP, α-1 acid glycoprotein; * significant difference compared with the control group

In the 40% of study group cats, ESR values were above the RI (Table 1). The percentage of outliers was 62.5% (5/8) in FIV^+^ cats, 42.9% (3/7) in *Li*^+^FIV^+^ cats, and 30% (6/20) in *Li*^+^ cats. The ESR values correlated negatively with RBC, haemoglobin (Hgb), haematocrit (Hct), albumin, A/G ratio and TIBC, and positively with mean corpuscular volume (MCV), mean cell haemoglobin (MCH), total proteins (TP), total globulins, IgG, IgM, Hp and α_1_-globulins and γ-globulin measures (Table 3). Higher measures of ESR were found in study group cats with hypoalbuminaemia (*n* = 4, *P* < 0.001), hyperglobulinaemia (*n* = 2, *P* = 0.019), A/G ratio below the RI (*n* = 5, *P* = 0.001), α_2_-globulins (*n* = 2, *P* = 0.026) and γ-globulins (*n* = 18, *P* < 0.001) above the RI. Additionally, the prevalence of cats with ESR above the RI was significantly higher in cats with hypoalbuminaemia (*n* = 4/4; *P* = 0.022; OR = 0.06; 95% CI = 0.002–1.16) or increased γ-globulins concentrations (*n* = 12/18; *P* = 0.002; OR = 14; 95% CI = 2.37–82.7).

**Table 3:** Correlation matrix between erythrocyte sedimentation rate values and the markers of inflammation considered in the cohort of 45 cats

Parameters	*r*_s_	*P* value
CBC		
Red blood cells	− 0.761	< 0.001*
Haemoglobin	− 0.663	< 0.001*
Hematocrit	−0.697	< 0.001*
MCV	0.450	0.002*
MCH	0.350	0.018*
MCHC	− 0.179	0.240
RDW	− 0.101	0.506
White blood cells	0.115	0.450
Neutrophils	0.186	0.221
Lymphocytes	− 0.081	0.595
Monocytes	0.263	0.080
Eosinophils	−0.028	0.853
Platelets	0.072	0.636
NL	0.101	0.546
ML	0.047	0.777
PL	−0.086	0.640
SBP		
IgG	0.634	< 0.001*
IgM	0.340	0.028*
Fe	−0.065	0.675
TIBC	− 0.375	0.012*
SAA	−0.117	0.444
Haptoglobin	0.365	0.019*
AGP	0.279	0.116
Total proteins	0.509	0.0004*
SPE		
Albumin	−0.667	< 0.001*
α_1_-globulins	0.356	0.018*
α_2_-globulins	0.265	0.082
β_1_-globulins	0.192	0.212
β_2_-globulins	0.212	0.167
γ-globulins	0.696	< 0.001*
Globulins	0.693	< 0.001*
A/G	−0.764	< 0.001*

CBC, complete blood count; MCV, mean corpuscular volume; MCH, mean corpuscular haemoglobin; MCHC, mean corpuscular haemoglobin concentration; RDW, red cell distribution width; NL, neutrophils-to-lymphocytes ratio; ML, monocytes-to-lymphocytes ratio; PL, platelets-to-lymphocytes ratio; SBP, serum biochemical profile; IgG, immunoglobulin G; IgM, immunoglobulin M; Fe, iron; TIBC, total iron binding capacity; SAA, serum amyloid A; AGP, α-1 acid glycoprotein; SPE, serum protein electrophoresis; A/G, albumin-to-globulin ratio; *r*_s_ Spearman’s rho; * significant correlation

### Comparisons between control and study group cats

In the study group, the values of ESR (*P* = 0.005), white blood cells (WBC) (*P* = 0.020), neutrophils (*P* = 0.012), IgG (*P* = 0.012), total globulins (*P* = 0.002), Hp (*P* = 0.003), total proteins (*P* = 0.004), α1-globulins (*P* = 0.033) and γ-globulins (*P* = 0.002) were significantly higher. In contrast, Hct concentrations (*P* = 0.036) and A/G ratio values (*P* = 0.004) were significantly lower (Table 2). The number of the study group cats with values above or below the RIs can be inferred from Table 1. The prevalence of γ-globulins concentrations above the RI (18/34) was significantly higher in the study group cats (*P* = 0.003; OR = infinity; 95% CI = 2.542 to infinity).

### Comparisons between *L. infantum*-positive and control group cats

Outlier values of *L. infantum* antibody-positive cats were not statistically different from the values of the control group cat. However, values of ESR (*P* = 0.031), leucocytes (*P* = 0.013), neutrophils (*P* = 0.011) and haptoglobin (*P* = 0.019) were significantly higher compared with the control group (Table 2). Although SAA concentrations resulted in significantly higher (*P* = 0.013) values in the control group, all values were within the RI in both groups (Table 2).

### Comparisons between FIV-positive and control group cats

The values of ESR (*P* = 0.016), total globulins (*P* = 0.002), IgG (*P* = 0.004), haptoglobin (*P* = 0.038), total proteins (*P* = 0.0003), α_1_-globulins (*P* = 0.023), α_2_-globulins (*P* = 0.043) and γ-globulins (*P* < 0.001) were significantly higher in FIV^+^ cats compared with the control group, and the A/G ratio (*P* = 0.004) was significantly lower (Table 2). The percentage of FIV^+^ cats with increased α_1_ -globulins (*P* = 0.007; OR = infinity; 95% CI = 2.340 to infinity) and γ-globulins (*P* = 0.002; OR = infinity; 95% CI = 3.807 to infinity) was statistically higher compared with the control group.

### Comparisons between cats antibody-positive to *L. infantum* and FIV and control group

The values of ESR (*P* = 0.007), total proteins (*P* = 0.0002), total globulins (*P* = 0.001), IgG (*P* = 0.002), IgM (*P* = 0.026), haptoglobin (*P* = 0.004), α_1_-globulins (*P* = 0.042), β_1_-globulins (*P* = 0.04), γ-globulins (*P* < 0.001) and NL (*P* = 0.042) were significantly higher in cats with dual antibody positivity compared with the control group, while A/G (*P* = 0.002) and Fe (*P* = 0.025) values were significantly lower (Table 2). Among cats with dual antibody positivity, the prevalence of cats with γ globulins value above the RI (6/7) was significantly higher compared with the control group (*P* < 0.001; OR = infinity; 95% CI = 5,010 to infinity) and the gammopathy was polyclonal.

## Discussion

This study showed that cats antibody-positive to *L. infantum* and/or FIV have higher ESR values than healthy antibody-negative cats, and 40% of them have values above the RI obtained from control group cats. We investigated ESR in cats infected by *L infantum* and FIV because in endemic areas cats with FIV have a higher risk for *L. infantum* infection, and in turn the co-infection is a risk factor for the development of FeL [7, 13]. Feline leishmaniosis and FIV infections both have a chronic course with clinical pathological changes reflecting chronic inflammation [13, 45]. The ESR is an inflammatory index due to the fact that the sedimentation of erythrocytes suspended in their plasma is faster in case of inflammatory conditions [33]. Dysprotidaemia associated with inflammation is one causative factor for the increased speed of RBC aggregation that precedes their sedimentation, and the physical mechanism of this process is still debated [46]. Hypoalbuminaemia, increases of positive APPs and immunoglobulins (IgM and IgG) occur together in inflammatory conditions, and contribute to elevate the speed of ESR [47]. This pathomechanism explains both the positive and negative correlations found between ESR and negative (albumin, albumin-to-globulin ratio and the TIBC) and positive (total globulins, γ-globulins, IgG, IgM, α_1_-globulins and haptoglobin) markers, respectively. For the same reason, a higher prevalence of ESR values above the RI has been detected in cats with hypoalbuminaemia or hypergammaglobulinaemia, and cats with hypoalbuminaemia, hyperglobulinaemia, low A/G, and increased α_2_-globulins and γ-globulins had significantly higher ESR measures. Changes in these parameters are influenced by inflammation, which in turn contributes to increasing the speed of ESR.

An additional cause for the increased speed of ESR is the reduction of Hct as it changes the proportion among RBC and plasma. As expected, Hct, RBC and Hgb values were negatively correlated with ESR measures. Moreover, a positive correlation with some erythrocyte indices (MCV and MHC) were observed as seen in humans [47, 48]. Mild to moderate anaemia occurs in chronic inflammation, and it is reported in *L. infantum*-infected cats, while severe anaemia is more frequent in FIV-positive cats [15]. Three cats had mild (3L, 4LF) or moderate (4F) normocytic normochromic non-regenerative anaemia, and they had multiple abnormalities in markers of inflammation, including increased ESR values in two of them.

Interestingly, a higher prevalence of increased ESR values was found in the FIV^+^ cats and in those with dual infection. The *Li*^+^ cats had low antibody titres (≤ 2-fold dilutions above the cut-off) and a lower prevalence of clinical signs compared with *Li* ^+^ and *Li* ^+^ FIV^+^group. Therefore, they may be in an early stage of the infection or be animals with a non-progressive form of infection. Longitudinal studies are needed to understand the dynamics of *L. infantum* infection in cats and markers of inflammation—including ESR—are useful to know for supporting prognostic evaluations. Based on the physical examination and CBC findings, the course of FIV infection was not advanced in studied cats as none of them suffered from myelosuppression, opportunistic infections or neoplasia [49]; however, their health status was in general more compromised compared with cats of *Li* ^+^ group when evaluated at physical examination (Table 1). Clinical findings observed in the study group were manifestations quite commonly reported in cats with oral diseases (34.2%; 12/35) and with enlarged lymph nodes (31.4%; 11/35) being the more prevalent, similar to a previous study in cats from the same area where outdoor cats had higher prevalences of oral disease and enlarged lymph nodes compared with indoors [50]. However, lymph node enlargement was significantly more prevalent in FIV^+^ and FIV^+^ *Li*^+^ cats. Donato et al. [50] investigated clinical epidemiological findings in cats studied for feline morbillivirus and the co-infection with FIV [51]. They found lymph node enlargement in about 30% of cats, but it was significantly correlated with the co-infection with FIV [47]. These previous field data and the present findings are explained by observations from a long-term experimental study that evidenced lymphoid hyperplasia more than five years after the experimental infection in monitored cats [52]. Before the terminal phase of FIV infection, most cats have a slow and progressive decrease in CD4^+^ T lymphocytes and show a dysregulation of immune response with chronic infections and polyclonal abnormal B-cell activation, both causing hyperglobulinaemia and hypergammaglobulinaemia also due to autoantibodies production [52–54]. In this study almost all FIV^+^ and *Li*^+^ FIV^+^ cats (14/15) had abnormalities at the SPE evaluation and in a significantly higher number of them increased γ-globulins (both groups) and α_1_-globulins (FIV^+^ group) levels were observed. The prevalence of ESR outliers was higher in FIV^+^ (62.5%) and *Li*^+^ FIV^+^ (42.8%) with respect to *Li*^+^ cats (30%). The difference was not significant but importantly this finding is in line with those from α- and γ-globulins.

An accurate evaluation of acute phase reaction in routine clinical pathological evaluation of cats is based on an APP profile which includes at least one positive major (SAA) and one positive moderate (Hp) APP [55]. Apart from SAA, Hp, and albumin as a negative APP routinely added in the SBP, we correlated ESR measures with many other blood parameters influenced by inflammation because they may be involved through different pathomechanisms causing their upregulation or depletion from blood, have different biological properties and kinetics, not completely known in cats [23]. We found increased values of at least one of the positive (SAA, AGP and Hp) and negative (albumin, TIBC, and Fe) AP markers studied in overall 15 cats. Six of them had from two to four abnormal AP marker values, and they were FIV^+^ (*n* = 3) and *Li*^+^ FIV^+^ (*n* = 3) cats, confirming that a more complex and probably multifactorial pathomechanism of inflammation may exist in FIV infection and co-infection with *L. infantum*. The AGP is an α_1_-globulin, but it was found to be elevated (912 μg/mL) in only one studied cat (3L), which had no signs at physical examination but mild non-regenerative anaemia, increased γ-globulins (23.7 g/L) and ESR (55 mm/h) values. No cats had abnormal TIBC values, including cats with low concentrations of Fe (*n* = 8) or albumin (*n* = 4). However, this parameter can be unfortunately found within reference range both in cases of iron depletion and sequestration [26]. Among the positive APPs studied, only haptoglobin showed a moderate correlation with ESR, with increased values in five cats (18L, 2F, 5F, 2LF, 5LF). Haptoglobin is an α_2_-globulin synthesised in the liver and well known for binding free Hgb, which is a by-product of hemolysis with pro-inflammatory activity [56]. However, increased α_2_-globulin values were seen only in cats 5F and 5LF. Immunoglobulins M and G were higher in FIV^+^ (IgG) and *Li*^+^ FIV^+^ (both IgM and IgG) cats compared with control cats, and values above RI were found only in cats with dual infections (LF1, LF5, LF6).

Uva et al. [37] found in cats with chronic kidney disease (CKD) increased values of both ESR and SAA compared with the healthy control cats, but they did not find the correlation between the two parameters [34]. In fact, they reported that ESR was significantly elevated in more advanced International renal interest society (IRIS) stages of CKD (IRIS stage 3 and 4) compared with SAA. In this study, six cats had CKD (Table 1) with four IRIS stage 1 cats (9L, 11L, 5F, 4LF), one stage 2 (4F) and one stage 3 (5LF) cats diagnosed. Elevated ESR values were measured in cats 9L, 4F, 5F and 5LF, while increased concentrations of SAA were only in cats 5F and 4LF. The presence of FIV and/or *L. infantum* infections in these cats and the limitations due to a small number of observations do not allow to speculate about the role of CKD in these abnormalities. Moreover, the ESR values (median = 13.5; 25th–75th percentile = 9.75–24 mm/h) of healthy control cats were lower (13.5 mm/h) in this study compared with Uva et al. [37] (median = 30.0, 25th–75th percentile = 16.0–37.0 mm/h) [34]. Both control groups included only ten adult individuals, which is a limitation. Extensive studies considering demographic variables are required to assess RI of ESR. Apart the study of Uva et al. [34, 37], no other data are available about ESR values in cat diseases and, despite the limitations from the small number of cats studied, we think that data obtained in the present study demonstrated that ESR values measured in EDTA blood with a POC device can be used as marker of inflammation in cats with the infectious and clinical conditions considered. Investigations have to be extended to obtain more robust results and perform multivariate analyses. There is a great need to have reliable, automated and easily accessible POC devices for clinical pathological investigations, and even more so to obtain these data with the smallest volume of blood collected from cats.

## Conclusions

This study found that ESR measured in EDTA blood with an automated device is a marker of inflammation in cats with *L. infantum* and/or FIV infections. Cats with FIV or dual infections have a more severe inflammatory condition, as shown by results from a pool of markers of inflammation never simultaneously assessed in the feline infections studied and in other cat diseases.

### Supplementary Information

**Additional file 1: Table S1** Reference intervals and measure units (MU) of complete blood count, serum biochemistry profile, serum protein capillary electrophoresis and urinary parameters that were statistically evaluated. The name of the analysers and the techniques used and the number of cats evaluated are reported. Apart from urinary parameters, values were considered outliers when exceeded more than 10% the upper (increased values) or the lower (decreased values) limit of the interval

## Data Availability

The data supporting the findings of the study must be available within the article and/or its supplementary materials, or deposited in a publicly available database.

## Abbreviations

A/G: Albumin-to-globulin ratio
AGP: α-1-Acid glycoprotein
ALP: Phosphatase alkaline
ALT: Alanine aminotransferase
APP: Acute phase protein
BCRs: Blood cell ratios
BUN: Blood urea nitrogen
CanL: Canine leishmaniosis
CBC: Complete blood count
CKD: Chronic kidney disease
CI: Confidence interval
Cr: Creatinine
EDTA: Ethylendiaminotetracetyc acid
ELISA: Enzyme-linked immunosorbent assay
ESR: Erythrocyte sedimentation rate
Fe: Serum iron
FeL: Feline leishmaniosis
FeLV: Feline leukemia virus
FIV: Feline immunodeficiency virus
FIV^+^: Antibody-positive to feline immunodeficiency virus
Hct: Hematocrit
Hgb: Hemoglobin
Hp: Haptoglobin
IFA: Immunofluorescence antibody assay
IFN: Interferon
IgG: Immunoglobulin G
IgM: Immunoglobulin M
IRIS: International renal interest society.
*Li*^+^: Antibody-positive to *Leishmania infantum*
*Li*^+^ FIV^+^: Antibody-positive to *Leishmania infantum* and feline immunodeficiency virus
MCH: Mean cell hemoglobin
MCHC: Mean corpuscular hemoglobin concentration
MCV: Mean corpuscular volume
ML: Monocyte-to-lymphocyte ratio
MoI: Markers of inflammation
NL: Neutrophil-to-lymphocyte ratio
PL: Platelet-to-lymphocyte ratio
POC: Point of care
OR: Odds ratio
RBC: Red blood cells
RDW: Red cell distribution width
RI: Reference interval
SAA: Serum amyloid A
SBP: Serum biochemical profile
SDMA: Symmetric dimethylarginine
SPE: Serum protein electrophoresis
TIBC: Total iron binding capacity
TP: Total proteins
UPC: Urine protein to creatinine ratio
WBC: White blood cells
