# Supplementary material for: The erythrocyte sedimentation rate and other markers of inflammation in cats tested for Leishmania infantum and feline immunodeficiency virus antibodies
Source: Parasit Vectors. 2024 Jul 30;17:324. doi: 10.1186/s13071-024-06396-1 (PMC11290220; doi:10.1186/s13071-024-06396-1)
Supplement: Supplementary file 1 — Additional file 1: Table S1 Reference intervals and measure units (MU) of complete blood count, serum biochemistry profile, serum protein capillary electrophoresis and urinary parameters that were statistically evaluated. The name of the analysers and the techniques used and the number of cats evaluated are reported. Apart from urinary parameters, values were considered outliers when exceeded more than 10% the upper (increased values) or the lower (decreased values) limit of the interval [file 13071_2024_6396_MOESM1_ESM.docx]

**Table S1.** Reference intervals and measure units (MU) of complete blood count, serum biochemistry profile, serum protein capillary electrophoresis, and urinary parameters were statistically evaluated. The name of the analysers and the techniques used, and the number of cats evaluated are reported. Apart from urinary parameters, values were considered outliers when exceeded more than 10% the upper (increased values) or the lower (decreased values) limit of the interval.

| Parameters | Number of cats evaluated | Analyser and reference Intervals | | |
| --- | --- | --- | --- | --- |
| Complete blood count (MU) |  | Siemens Advia 2120 | ProCyte Dx | Futurlab DF50 |
| Red blood cells (M/μL) | 45 | 5.85-12.21 | 5.89-13.42 | 4.16-11.22 |
| Hemoglobin (g/dL) | 45 | 8.2-18.04 | 8.82-17.8 | 7.7-16.8 |
| Hematocrit (%) | 45 | 27.7-49.0 | 30.3-52.3 | 26-47 |
| MCV (fL) | 45 | 33.4-56.4 | 32.3-58.4 | 34.2-59.4 |
| MCH (pg) | 45 | 11.3-18.7 | 10.62-19.03 | 10.62-19.8 |
| MCHC (g/dL) | 45 | 26.82-41.47 | 25.3-39.4 | 26.1-39.6 |
| RDW (%) | 45 | 12.33-20.57 | 13.5-29.7 | 14.4-25.3 |
| White blood cells (K/μL) | 45 | 4.23-19.14 | 2.6-18.7 | 4.95-21.45 |
| Neutrophils (K/μL) | 45 | 1.71-13.97 | 2.07-11.32 | 2.81-13.84 |
| Lymphocytes (K/μL) | 45 | 1.08-7.70 | 0.83-7.57 | 0.66-8.65 |
| Monocytes (K/μL) | 45 | 0-0.66 | 0-0.74 | 0-1.49 |
| Eosinophils (K/μL) | 45 | 0.90-1.98 | 0.15-1.73 | 0.05-2.12 |
| Platelets (K/μL) | 45 | 91.8-647.9 | 136-660 | 90-569.8 |
| Serum biochemical profile (MU; technique) |  | Beckman Coulter AU 5800 |  | |
| Immunoglobulin IgG (mg/dL; immunoturbidimetry^) | 42 | 200.7-1257 |  | |
| Immunoglobulin IgM (mg/dL; immunoturbidimetry^) | 42 | 24.3-165 |  |  |
| Iron (μg/dL; colorimetry^) | 44 | 49.5-176 |  |  |
| Total iron binding capacity (μg/dL; calculated method∞) | 44 | 198-464.2 |  |  |
| Serum amyloid A (μg/mL; latex agglutination°) | 45 | 0.09-3.3 |  |  |
| Haptoglobin (mg/dL; immunoturbidimetry^) | 41 | 3.6-156.2 |  |  |
| α-1 acid glycoprotein (μg/mL; ELISA∞) | 33 | 0-550 |  |  |
| Total proteins (g/L; colorimetric photometry^) | 44 | 52.2-93.5 |  |  |
| AST (IU/L; kinetic UV photometry^) | 44 | 11.7-53.9 |  |  |
| Alanine aminotransferase (IU/L; kinetic UV photometry^) | 44 | 0-88 |  |  |
| Alkaline phosphatase (IU/L; kinetic colorimetric photometry^) | 44 | 3.9-61.6 |  |  |
| Gamma glutamyl transferase (IU/L; kinetic colorimetric photometry^) | 44 | 0-3.3 |  |  |
| Total bilirubin (mg/dL; colorimetric photometry^) | 44 | 0-0.33 |  |  |
| Glucose (mg/dL; enzymatic UV photometry^) | 44 | 70.2-166.1 |  |  |
| Fructosamine (μmol/L; kinetic colorimetric photometry*) | 42 | 153-396 |  |  |
| Blood urea nitrogen (mg/dL; kinetic UV photometry^) | 44 | 31.5-83.6 |  |  |
| Creatinine (mg/dL; kinetic colorimetric photometry^) | 44 | 0.63-1.98 |  |  |
| Serum symmetric dimethylarginine (μg/dL; immunoturbidimetry≈) | 43 | 0-19.8 |  |  |
| Phosphorus (mg/dL; UV photometry^) | 44 | 2.34-7.04 |  |  |
| Serum protein capillary electrophoresis (MU) |  | Sebia Capillarys 3 Tera |  | |
| Albumin (g/L) | 44 | 26.76-49.29 |  | |
| α_1_-globulins (g/L) | 44 | 0.62-3.56 |  |  |
| α_2_-globulins (g/L) | 44 | 4.86-14.79 |  |  |
| β_1_-globulins (g/L) | 44 | 2.27-9.68 |  |  |
| β_2_-globulins (g/L) | 44 | 2.09-9.56 |  |  |
| γ- globulins (g/L) | 44 | 3.15-20.57 |  |  |
| Globulins (g/L) | 44 | 23.4-59.4 |  |  |
| Albumin-to-globulin ratio | 44 | 0.54-1.43 |  |  |
| Urinalysis |  |  |  | |
| Urine specific gravity | 25 | >1035 |  | |
| UPC^ | 21 | ≤ 0.4 |  |  |

Legend: UPC: urinary protein-to-creatinine ratio; ^: Beckman Coulter, Milano, Italia; ∞: calculated method (total iron^ + unsaturated iron binding capacity, Sentinel CH, Milano, Italia; °: VET-SAA, Eiken Chemical Co. Ltd., Tokio, Japan; ∞: Nextmune Laboratories, Wetherby, United Kingdome; ≈: Eurolyser Diagnostica,Salzburg, Austria.
